# Social hierarchy and the choice of metal recycling at Anyang, the last capital of Bronze Age Shang China

**DOI:** 10.1038/s41598-020-75920-x

**Published:** 2020-11-02

**Authors:** Ruiliang Liu, A. Mark Pollard, Qin Cao, Cheng Liu, Victoria Sainsbury, Philly Howarth, Peter Bray, Limin Huan, Bohao Yao, Yuting Fu, Jigen Tang

**Affiliations:** 1 School of Archaeology, University of Oxford, Oxford, OX1 3TG UK; 2 National Museums Scotland, Chambers Street, Edinburgh, EH1 1JF UK; 3 School of Cultural Heritage, Northwest University, Xi’an, 710069 China; 4 British Museum, London, WC1E 7JW UK; 5 Department of Statistics, University of Oxford, Oxford, OX1 3LB UK; 6 Department of Mathematics, University of Oxford, Oxford, OX2 6GG UK; 7 Center for Social Sciences, Southern University of Science and Technology, Shenzhen, 518055 China

**Keywords:** Geochemistry, Geology

## Abstract

Anyang, the last capital of the Chinese Shang dynasty, became one of the largest metal consumers in Eurasia during the second millennium BCE. However, it remains unclear how Anyang people managed to sustain such a large supply of metal. By considering the chemical analysis of bronze objects within archaeological contexts, this paper shows that the casting and circulation of metal at Anyang was effectively governed by social hierarchy. Objects belonging to the high elites such as Fuhao, particularly the bronze ritual vessels, were made by carefully controlled alloying practice (primary) using very pure copper, whereas the lower elites only had access to bronzes made by secondary alloying practice and copper with more impurities. Such contrasts allow scholars to identify those objects which are less likely to have been made by mixing and recycling, which has very important implications for the chemical and isotopic determination of provenance for future studies.

## Introduction

Metal is one of the earliest fully reusable materials discovered by human beings. Unlike other natural materials, such as stone (e.g., marble or jade), it can, in theory, be infinitely recycled^1^. Considering the comparative rarity of metal deposits on earth, this recyclability gives metal not just the acknowledged technological or functional engineering advantages, but also an extra dimension of economic, social, cultural and ritual significance^2^. For example, the rise of the Scandinavian Bronze Age was partially dependent on the recycling of imported objects in order to sustain the production of those objects in locally favoured shapes and styles^3^. Indigenous sources of metal were scarce, and so such imported objects were exploited as a source of raw material. In contrast, elite swords, which are commonly considered to be objects with high functional and symbolic value in broader Bronze Age Europe, contained very little recycled metal^4^. In later periods, where more textual information is available alongside the archaeological and archaeometric data, one can gain much more specific knowledge about the spiritual and religious power of the objects and how recycling can affect such powers^5^.

**Figure 1:**
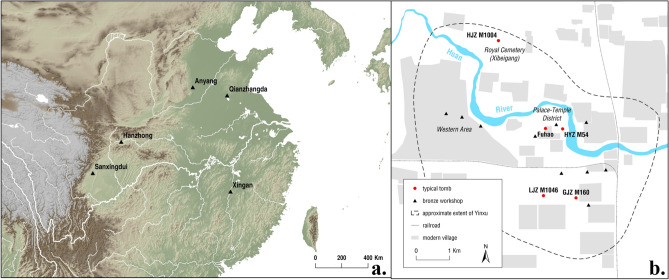
Locations of Shang sites in this article (**a**, produced by ArcGIS 10.8 (arcgis.com) with the elevation data from SRTM) and the plan of Anyang (**b**, modified from reference^19^).

Does the recycling of metals, which has been commonly encountered in many parts of Bronze Age Eurasia, also occur in China? If so, what social factors could affect recycling in China? In its early dynasties (Shang and Zhou, ca. 1600–221 BCE), large numbers of extraordinary bronze vessels were produced. They were employed in banqueting, offering food and drink for ancestor worship, signalling social status and materializing ritual activities^6–9^. A great deal of precious raw materials, including copper, tin and lead, were devoted to the production of these vessels, which were then deposited in the tombs of elite persons^8^. Crucially, many of these bronzes were cast with inscriptions, allowing them to be associated with specific elite individuals or lineages^10^. This also indicates that craftspeople were well aware of for whom these bronzes were being made. From a broader perspective, the preference for making bronze ritual vessels is a phenomenon unique to China. In many other parts of Bronze Age Eurasia, the most typical bronze products were weapons, tools and personal ornaments^11^. The broader issue is what strategy was implemented to ensure the sustainable supply needed to maintain metal production in Bronze Age Shang China (ca. 1600–1045 BCE), the first Chinese dynasty which shows overwhelming archaeological and textual evidence that huge quantities of metal were removed from the contemporary metal circulation and deposited in tombs? So far, the study of recycling in China is limited by the lack of contemporary written records. A few Eastern Zhou bronze vessels of the late Bronze Age, dating from 771 to 256 BCE, have inscriptions which refer to the melting down of weapons after battles for the casting of new ritual vessels. On the other hand, an Eastern Zhou book, ‘Rites of Zhou’, records six specific alloying recipes for different object types, none of which mentions recycled metal^12^. Anyang, the last capital of dynastic Shang China (ca. 1250–1045 BCE, Fig. 1), offers an excellent case study for these issues, partly owing to the quantity of metal found in numerous elite tombs, but also because social hierarchy is clearly demonstrated by burials^6,8,13^. The remarkable difference in social status and individual wealth is not only indicated by the quality and quantity of metal objects, but also by inscriptions on bronzes, by the numbers of human/animal sacrifices, by the size, positioning and orientation of the burial chamber, and by other associated material wealth (e.g., jade, ivory, white pottery, gold, and bone objects).

The scale of production at Anyang is vividly reflected by an astonishing 1.6 tonnes of metal discovered in Fuhao’s tomb alone—the only intact top royal burial so far excavated at Anyang- yet she was only one of the royal consorts to King Wu Ding, and was by no means the most important figure buried in Anyang^14^. Anyang can be divided into four chronological phases (Yinxu I–IV: Supplementary Fig. S1 and Table S1^15^) and all the kingly tombs are located in Xibeigang. Though repetitively looted, they retain many indications that they were also richly furnished, and were up to seven times larger than Fuhao’s tomb (Supplementary Table S2). This suggests that the quantity of metal discovered in her grave only represents the tip of the iceberg. To sustain such an immense amount of metal production, there must have been a highly organised system at Anyang which was capable of not only obtaining metals from distant regions, over 500 km away^8,16,17^, but also of producing a large variety of objects and fulfilling the different requirements of many people.

This paper will place the alloying and impurity patterns of metal objects from Anyang within their social contexts, particularly focusing on the variation of alloying practices. It aims to demonstrate that the choice of recycling can be inferred from the metallurgical chemistry record in certain situations. Furthermore, we demonstrate that disentangling the choice of metal recycling at Anyang helps to understand the system underpinning the mass production and the flow of metal within this hierarchical society. Furthermore, identifying objects containing little or no recycled metal is also useful when considering provenance studies, since such objects are more likely to be traceable to source^18^.

## Alloying and impurity patterns of the high/low-elite metal assemblages at Anyang

Of the thousands of tombs discovered at Anyang, many were looted in antiquity. Only a small number of tombs with rich bronzes (over 100 pieces) were found intact and some of their bronzes (both weapons and ritual vessels) have compositional analyses: Fuhao, M1004, M54, M160 and M1046 (Supplementary Table S2). Large numbers of weapons (n = 941) were uncovered from M1004, a kingly tomb which was heavily looted in antiquity, and which is radiocarbon dated to Anyang Phase II (1243 BCE–1157 BCE, 95.4% probability, Supplementary Fig. S1). In addition to the remarkable quantity of bronzes found within these tombs, they had other evidence of material wealth (e.g., jade or ivory), human/animal sacrifices and large sized tomb chambers with more sophisticated structures (up to four tomb passages). From the inscriptions on some of the high-quality bronze ritual vessels, individuals have been identified by correlating with the same names in the recordings on oracle bones, such as Fuhao, a Shang queen^14^. There are also tombs with much fewer bronzes found in the Western Area, a massive cemetery with thousands of tombs dated to all four phases at Anyang, identified as cluster burials of lineage members^20^. As demonstrated by the burial contents, in particular the quantity and quality of bronzes, numbers and types (humans or dogs) of sacrifices, along with the size, orientation and structure of the tomb^13,20^, these tombs belong to individuals with lower social status in comparison to those five tombs discussed above. In order to address the question proposed in this article and facilitate the discussion below, we refer to the five tombs with remarkable numbers of bronzes (over 100) as the ‘high elite tombs’, and those from the Western Area are categorized as ‘low elites’. The concept of the high/low elites in this paper is primarily built upon established studies of Shang social hierarchy based on mortuary remains^13,20^. It offers a simple convenient terminology and framework which includes a wide range of other factors for these individuals, such as social networks, gender, origins, family backgrounds, personal abilities or professions. Nevertheless, since the fundamental objective is to capture the broad pattern of the metallurgical practices, it is necessary to apply this generalisation on the spectrum of the complicated social hierarchy at Anyang, in order to facilitate the discussion below. These individuals with their bronzes not only represent a fraction of the overall metal consumption, but are also the only examples with fully published chemical compositional data so far available.

In an exercise such as this, where chemical analyses from several different authors and analytical methods are combined, an understanding of the quality of the data is essential to build the archaeological narrative. Whilst the analytical instruments and conditions are sometimes recorded in detail in the literature, it is still rather challenging to carry out a thorough quality assessment without reports on primary and secondary standards (Supplementary Table S5). What we are looking for here is only the broad patterns, based on the largest possible database available across different metal assemblages, rather than any detailed case study (e.g., a single object), since neither the chemical analysis nor the excavation information published so far supports such individual investigations. Furthermore, these patterns are statistically robust and reproducible in terms of the specific archaeological context, offering a crucial point of departure for the discussion presented here.

**Figure 2:**
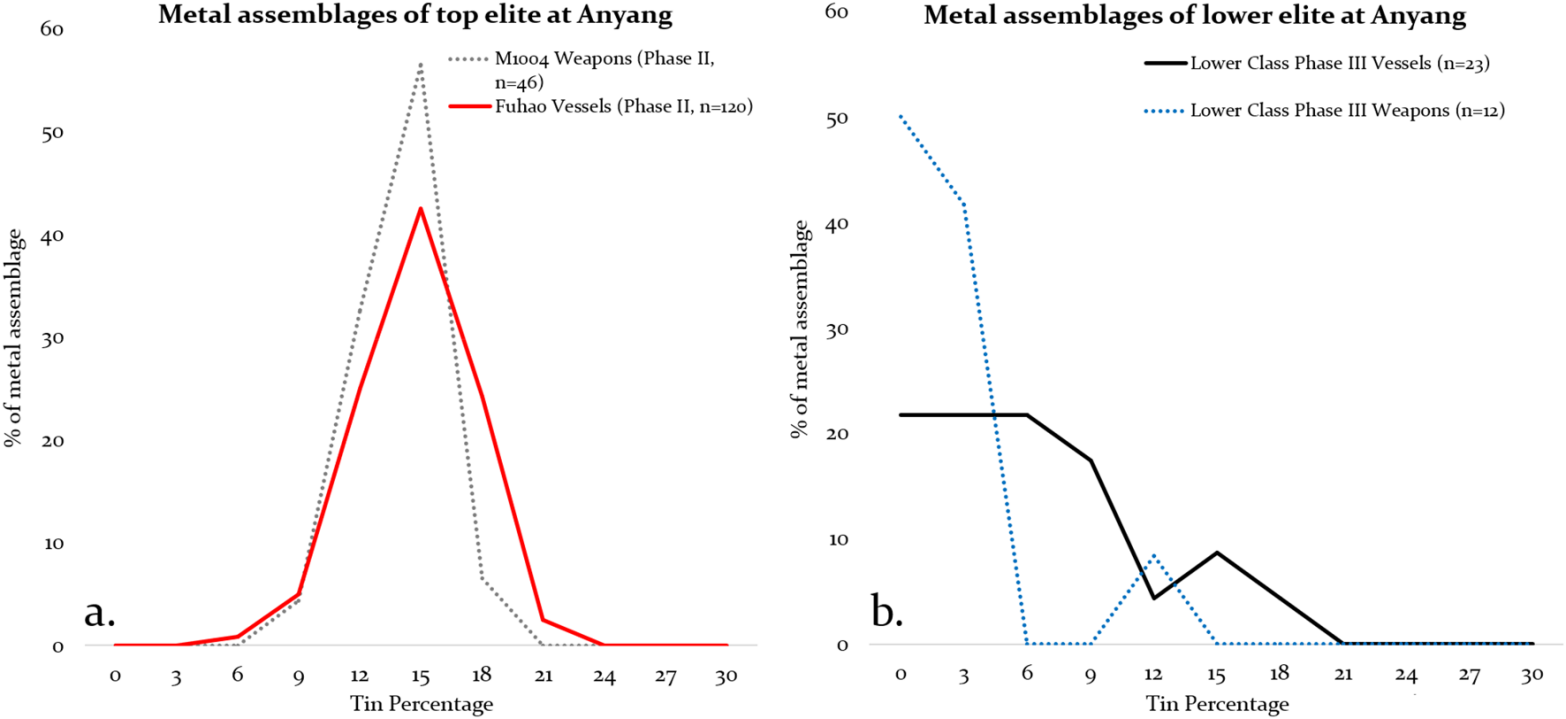
Tin distributions of selected high elite and low elite assemblages at Anyang.

The alloying elements associated with copper in Bronze Age Central China are primarily tin and lead. By looking at the distribution of tin and lead in an assemblage of metal objects, it is possible to identify some distinct patterns between high and low elites. In this study, the term assemblage is defined by the tomb in which the objects are found. Using Fig. 2 as an illustrative example, one can clearly see two basic patterns of tin distribution. For the bronze ritual vessels of Fuhao and the weapons found in M1004, both of which are defined as the high elites, they show a regular symmetric distribution (approximately normal) around a specific value (Fig. 2a). This can be interpreted as being the result of deliberate control of the alloying process, with a single target composition in mind, which is referred to here as primary alloying practice. Considering the large sample analysed (n = 46 and 120, respectively), this further testifies to the overall compositional consistency of metals available to the high elites during Anyang Phase II. This process of primary alloying practice seems to not only refer to the percentage of tin or lead added, but also to the quality of the copper, which is chemically very pure, suggesting that it is highly refined. Here we refer to this type of copper as primary copper (like primary alloying), which contains very little impurities (see below). Furthermore, the term primary copper differs from smelted/prime copper, which conventionally means the fresh copper from the smelting process, typically bearing a suite of impurity elements in much higher percentages.

In contrast with the primary alloying practice, both the vessels and weapons of the low elites in Phase III (1197 BCE–1108 BCE, 95.4% probability) present a typical skewed pattern, or an asymmetric distribution, as shown in Fig. 2b. This could be the result of several processes, such as objects being made from different alloying recipes (implemented by different foundries or requested by various patrons), or mixing and recycling of finished objects/copper ingots, leading to less well controlled and (generally) diluted (lower) alloy compositions. This set of circumstances is defined here as a secondary alloying practice. Given the fact that people buried in the Western Area were of much lower status than a queen (Fuhao) and king (the occupant of M1004), it is reasonable to suggest that they did not have the same access to objects made from primary alloying practice. In this light, mixing and recycling would more likely have taken place for these low elites (see below).

**Figure 3:**
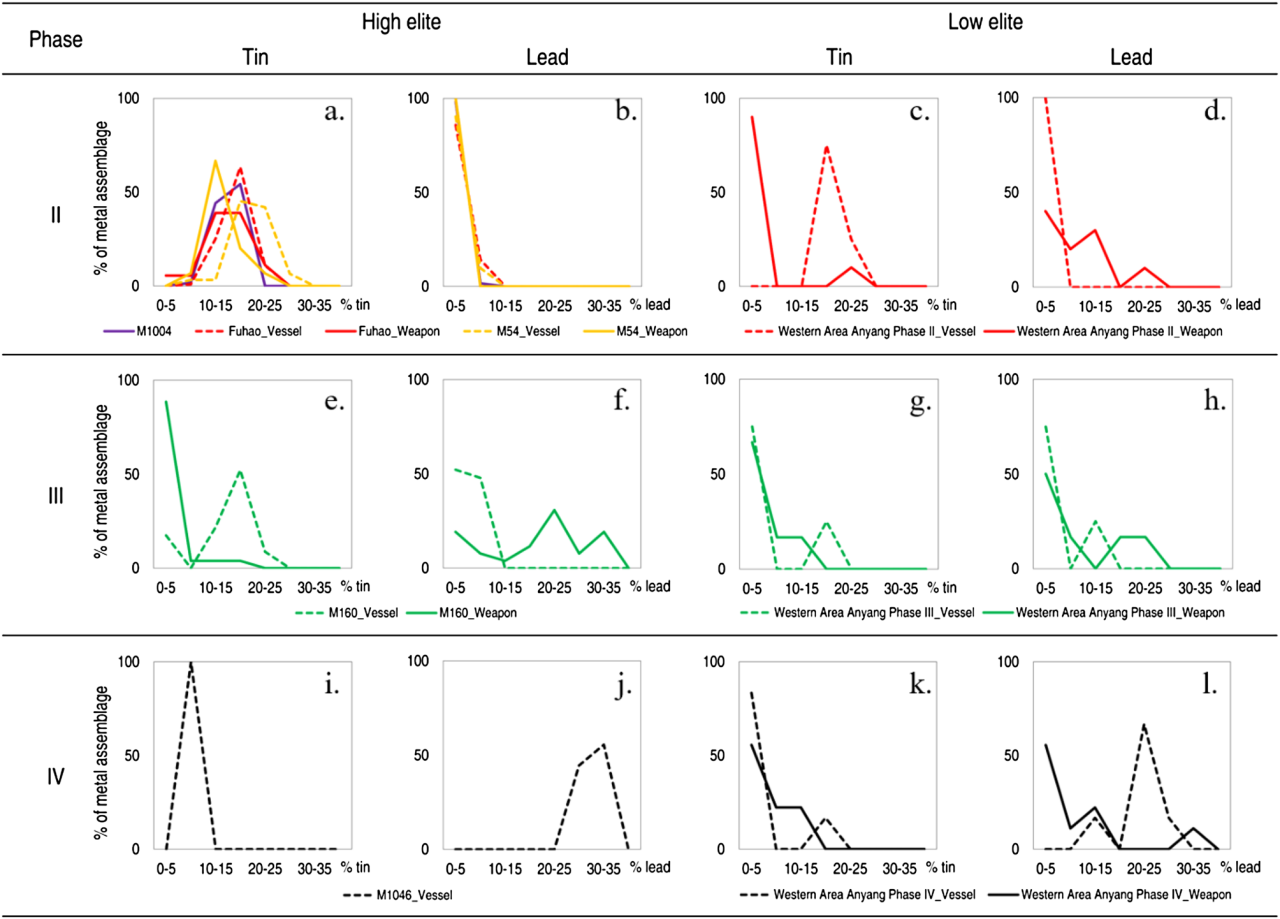
Variation of alloying elements at Anyang.

The contrast between primary and secondary alloying can be seen in a number of other metal assemblages of high- or low-elites at Anyang in Phases II to IV (Fig. 3). All the distribution patterns of tin and lead in the weapons of M1004, vessels and weapons of Fuhao and M54, vessels of M160 and M1046 indicate primary alloying. In sharp contrast, objects belonging to the low elites buried in the Western Area are more likely to follow the pattern of secondary alloying. Almost identical to the patterns from the weapons and vessels of the Western Area of Anyang Phase III (Fig. 2b), those dated to Phases II and IV also show a typical skewed distribution. Kernel density estimation renders further support for this by demonstrating that the range of tin or lead in the high elite metals is always narrower than the equivalent in the low elites (Supplementary Fig. S2 and Tables S3–S4).

It becomes even more complex when a crucial change in alloying compositions took place in Phase III, changing from high-tin to high-lead recipes, particularly for the high-elite bronze objects. The prevailing argument is the widespread shortage of tin since the Anyang Phase III, even for the production of high elite objects. Therefore, lead became the obvious substitute^21^. Vessels in M1046, a high-elite tomb with 123 bronzes dated to Phase IV, contain a striking amount of lead, almost 25% (in absolute terms) more than in all the vessels or weapons dated to Phase II, which are high-tin bronzes (tin ~ 12–18%). More interestingly, among bronzes in M160, a high-elite tomb of Phase III, one can see a sharp difference in alloying between vessels (high tin and low lead) and weapons (low tin and high lead, Fig. 3e–f). One common interpretation is that these weapons with high percentages of lead (over 20%) were made solely for burial rather than for functional purpose, which could have been the result of changing ritual beliefs^22,23^. On a closer look at these so called ‘weapons for burials’, particularly *ge* (dagger-axes), the most common Shang weapon type from a typological perspective, they all belong to a particular type—dagger-axes with curved tang and no base^24,25^. Based on material characteristics of these thin and lightweight weapons with high lead content, an alternative explanation is that they were effective for ceremonial display (with a specific grey-silvery colour^26^) and martial performance, showcasing martial hegemony and power^27^.

Under this broad picture of the metal objects from tombs of low/high elites, a few exceptions arise. It is noticeable that the distribution of lead in M1004, Fuhao, M54 are clearly skewed towards the bin 0–5%, which requires further explanation (Fig. 3b). The benefit of adding an appropriate amount of lead (~ 2–5%) would be to lower the melting temperature and add fluidity to the molten metal. Whilst low lead contents in a few objects very likely originate from native impurities associated with copper ore (e.g., Pb < 0.5%), in this context, lead is detected above this impurity level in the majority of objects from the overall assemblage, suggesting that it was intentionally added, but not in as large or as controlled quantities as the tin. In the Western Area assemblages, the vessels dated to Phases II and IV are also exceptional. Their distributions of tin or lead show primary alloying patterns. The Kolmogorov–Smirnov test shows no significant difference in the tin level in the bronze ritual vessels between low- and high-elites assemblages during Phase II (Supplementary Table S3). This might be explained by the lack of data for the low-elite ritual vessels (n = 4). However, if further data support this, then it suggests that during Phase II the supply of tin was sufficiently abundant to support the production of ritual vessels for both high and low elites, despite the enormous quantities that must have been required. Their alloying patterns are those of the high elites, indicating better control of the alloying than those made directly for low elites in subsequent phases. If primary alloying was a technique exclusively reserved for the production of high elite bronzes, one possibility for the low elite assemblage in Phase II containing a primary alloying signal is that they were initially made according to high-elite recipes and subsequently given by the high elites to the low elites (e.g. as gifts or rewards, see below). Interestingly, a similar explanation cannot be applied to the vessels of the Western Area in Phase IV, which, although visually conforming to a primary alloying pattern, appear statistically different from high-elite metal objects (Supplementary Table S4). This might be attributed to the admittedly small number of data obtained for this phase, rendering the comparison imprecise. Though the weapons from Western Area Phase IV mostly indicate a secondary alloying practice, not all objects can necessarily be considered as completely recycled or containing recycled metal^28^. For instance, six out of ten samples contain tin around 7–14% and lead below 5% (similar to those in the tomb of Fuhao). These weapons can be considered to be highly functional in battles. If they are indeed made by recycling, then the only explanation would be recycling from a single well-made object without mixing with other sources. A further four weapons contribute to the tail of the skewed pattern (secondary alloying), with no tin but 3%, 8% and 12% lead or only 4% tin (with no lead). Such an alloying pattern appears less well controlled and would result in less functional weapons in practical battles, compared to the high elite weapons. In this sense, these weapons could be made by different foundries with perhaps different recipes, with low-quality metals for different purposes (e.g., burial) and the probability of involving mixed or recycled metal in their production is also higher.

**Figure 4:**
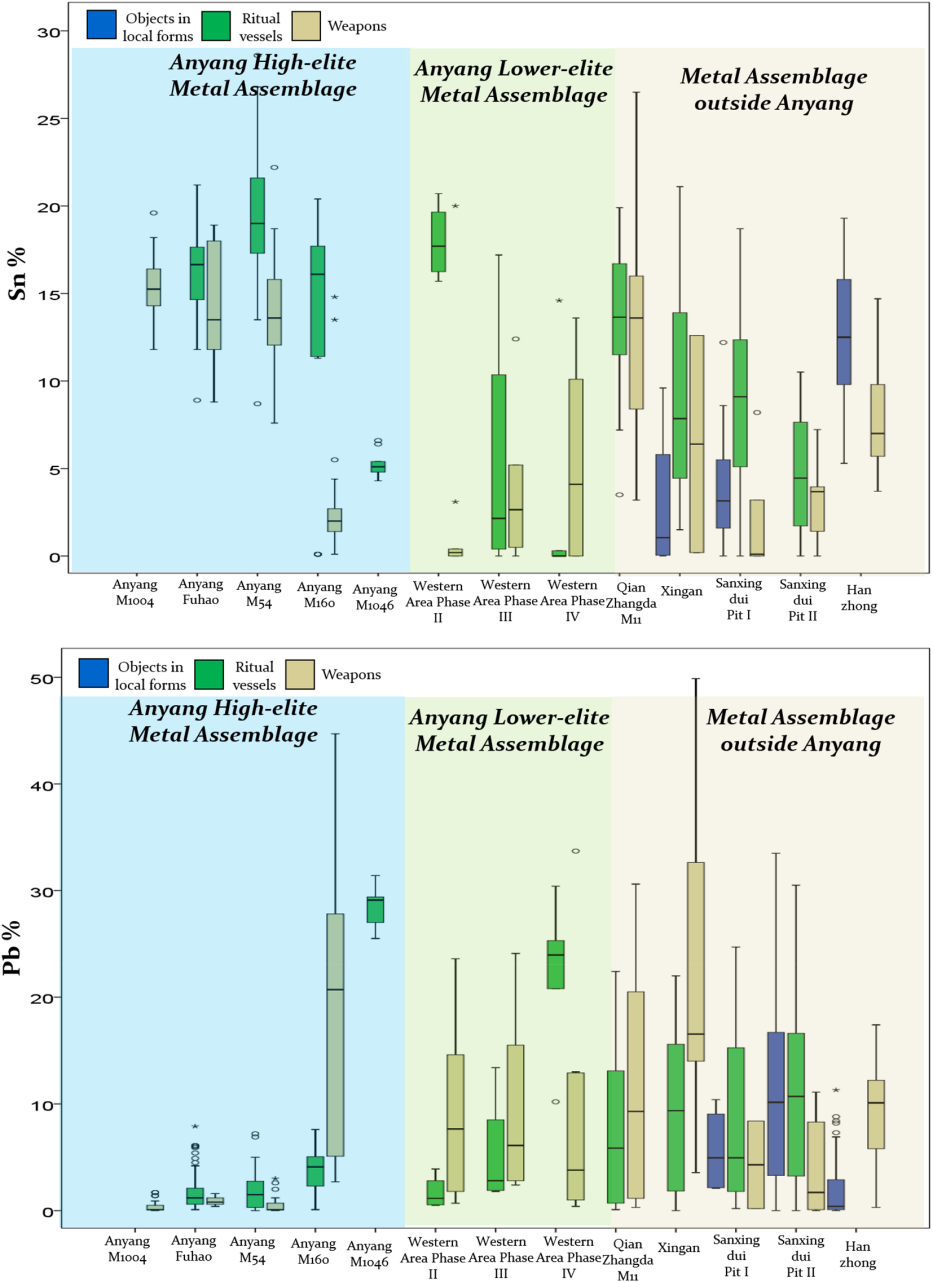
Summary of alloying pattern in the key metal assemblage during the Anyang period.

Figure 4 illustrates a broader picture by including multiple major metal assemblages from across Bronze Age China. These assemblages are also defined as objects from the same archaeological context, such as an individual tomb, or a single ceremonial pit^29,30^, and are dated to approximately the same time as the Anyang material. All of them show alloying patterns similar to those of the low-elites at Anyang. The only metal assemblages showing primary alloying practice are those of the Anyang high elites. This suggests that the manufacturing process for the high elite metals was not only unique in Anyang, but was also not replicated anywhere else in Bronze Age Central China.

Whilst is true to say that large scale recycling of objects made from a ternary alloy (Cu–Sn–Pb) would tend to produce an assemblage with an average composition, and with a distribution profile tending towards a normal distribution, this appears unlikely in the context of Shang China. Such a process would require a large number of recycling events to homogenise the composition to the degree seen in the high elite assemblages, which appears unlikely in this context. The large variation in tin and lead shown in the majority of late Shang bronzes (Fig. 4) cannot be averaged out by mixing and recycling. Even if it did, the final percentage of tin and lead cannot be as targeted or well controlled as observed in the objects of high elites (e.g., weapons of M1004 or vessels of Fuhao, Fig. 2a). The most parsimonious explanation is that presented here—that these high elite objects represent primary manufacture from new materials.

Turning the argument around is, however, not necessarily correct. As with the Western Area at Anyang, the wide range of alloying distribution in the metal assemblages at Qianzhangda, Xingan, Sanxingdui and Hanzhong could be explained by a range of various possibilities. In addition to mixing and recycling, these assemblages might consist of objects made by different foundries or in different periods, or even different batches with possibly different supplies of metal sources, thus with different recipes.

**Figure 5:**
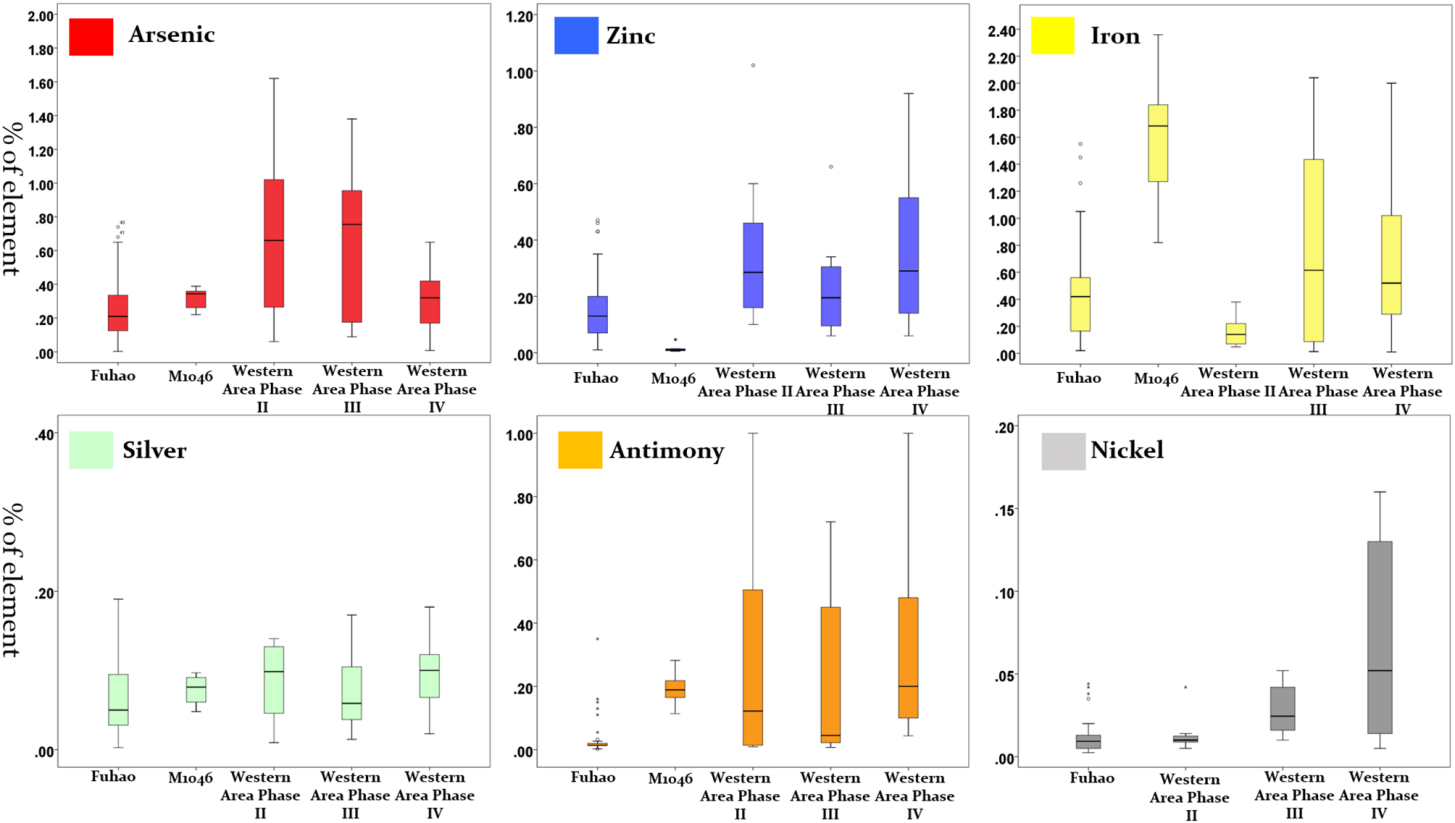
Comparisons of impurity variations between high- and low-elite metal assemblages.

Figure 5 summarizes the variations of impurity patterns (based on trace elements) for the currently available chemical data for the high-elite metal assemblages and those of low elites’ burials, divided according to chronology. Again, the objects from the high-elite assemblages (Fuhao and M1046), with lower levels and tighter distributions of multiple trace elements (arsenic, zinc, antimony, nickel), show a different pattern from those of the low elites. This suggests that either the copper is obtained from one or a limited number of very pure copper sources (perhaps from high grade malachite), or that it has been more carefully refined (thus the more easily volatilized elements such as arsenic, antimony or zinc are retained in lower concentrations). Otherwise, it would be very difficult to achieve such pure copper. One might wonder if the recycling of metal could also lead to the same trace element pattern. However, we think that the explanation involving refining appears more likely because, according to thermodynamics, it is very difficult to remove arsenic at levels below 0.3% simply by re-melting (or recycling). In spite of all other trace elements in the metal assemblage of M1046, which show very low concentrations, iron stands out as an anomaly. This is probably due to a combination of various factors, e.g. a specific ore source, smelting temperature, iron associated with galena, which are yet to be understood.

However, the variations of each impurity element in the metal assemblages from the low elites in the Western Area, in whichever phase, are dramatically greater than those in the high-elite data. This implies that these objects were made from one or more sources of less well-refined copper. Given the fact that the addition of tin or lead appears less well-controlled sometimes, it is reasonable to hypothesize that the copper used for the low-elite bronzes might not be well controlled either, and therefore that the possibility of mixing and recycling should be considered.

**Figure 6:**
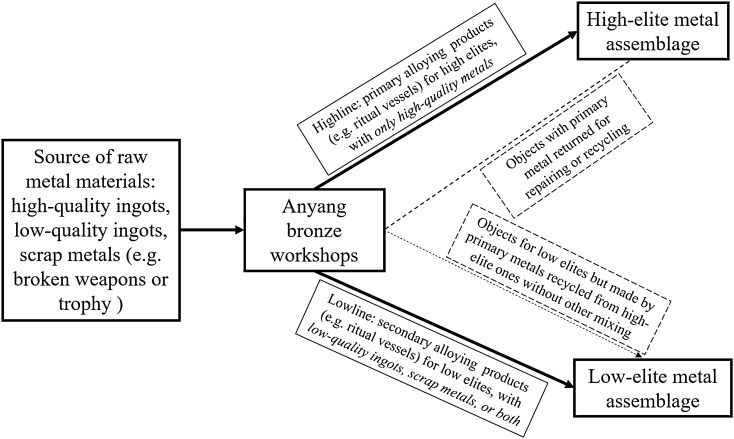
The two-line model for Anyang metal production.

The strategy for the sustainability of metal production at Anyang can be summarized as a two-line model (Fig. 6). The upper line corresponds to the production of metal objects by primary alloying using high-quality raw materials (e.g., highly refined copper and carefully added tin or lead), which can only be accessed by the high elites. However, some of these objects may end up in the tombs of low elites by processes such as top-down rewarding or gifting. The products from the lower line are for the low-elites, based on secondary alloying practices with relatively low-quality or recycled metals from multiple sources or foundries, making it nearly impossible for the craftspeople to precisely control the variation of tin or lead in the final objects. This two-line hierarchical model guarantees the requirements of people from different social classes for metal objects. Primary alloying practice with high-quality materials enables craftspeople to produce objects with targeted weight, colour, shape and decoration, whereas the needs for metal objects of the low elites can be fulfilled by lower-quality or recycled metals.

## Conclusions and future directions

Mixing and recycling of metals is always a choice in human societies. It appears that it was optional rather than compulsory at Anyang, and making such a decision involved not only technological or economic factors but also social considerations. The characterisation and comparison presented here show a variety of chemical patterns in the bronzes at Anyang, illustrating a highly complex metal production and consumption system. Yet the broad trends are rather clear. There was an important correlation between alloying practice, the selection of metal resources and the levels of social hierarchy. Two clearly distinguishable patterns can be recognised in both alloying and impurity data. The metal assemblages of the high elites are based upon a primary alloying practice and the use of cleaner copper, whereas those of the low elites are made by secondary alloying practices, revealing a fundamentally ‘hierarchical structure’ in the practices of alloying, mixing and recycling at Anyang. As with highly valued objects such as swords in Europe^4^, the objects owned by high elites at Anyang, particularly bronze ritual vessels, appear relatively unlikely to have been made by recycling or mixing, and are therefore more suitable for provenance studies. Recycling is not just driven by economic considerations, but also by many other demands. Here we contribute another case study in which the social, cultural and ritual value can affect the modes of production, circulation and consumption of metal in the Bronze Age. Considering the striking number of metal objects required by the entire Anyang society, it is not difficult to imagine that the metals deemed to be most ‘prestigious’ by Anyang people were exclusive to those elites with high status, with the low elites receiving lower quality metal. The synergy of the two broad categories allows Anyang to sustain its overall metal production on the one hand, and also manipulate the distribution of these objects to fulfil multiple social, political and ritual necessities of different groups of people on the other. More high-quality chemical analyses together with complete archives of the excavation context as well as the attributes of the objects in the future will enable scholars to investigate more details on the individual objects, the issue of mixing and recycling, as well as test the two-line model. Paired with other investigations, such as aDNA or stable isotopic analysis^31^ of the deceased, this will further illuminate the influence of social stratification on the circulation of metal objects at Anyang and beyond.

## Supplementary information

Supplementary Information 1.

Supplementary Information 2.
