# Supplementary material for: Social hierarchy and the choice of metal recycling at Anyang, the last capital of Bronze Age Shang China
Source: Sci Rep. 2020 Nov 2;10:18794. doi: 10.1038/s41598-020-75920-x (PMC7606620; doi:10.1038/s41598-020-75920-x)
Supplement: Supplementary file 2 — Supplementary Information 2. [file 41598_2020_75920_MOESM2_ESM.docx]

**Online Supplementary Material**

**Title:** Social hierarchy and the choice of metal recycling at Anyang, the Last Capital of Bronze Age Shang China

**Author**: Ruiliang Liu^1^^*^, A. Mark Pollard^1^, Qin Cao^2^, Cheng Liu^3*^, Victoria Sainsbury^1^, Philly Howarth^1^, Peter Bray^4^, Limin Huan^1^, Bohao Yao^5^, Yuting Fu^6^, Jigen Tang^7^

**Institute**: 1. School of Archaeology, University of Oxford, United Kingdom (OX1, 3TG)

2. National Museums Scotland, Chambers Street, Edinburgh (EH1 1JF)

3. School of Cultural Heritage, Northwest University, Xi'an, China (710069)

4. British Museum, United Kingdom (WC1E 7JW)

5. Department of Statistics, University of Oxford, United Kingdom (OX1 3LB)

6. Department of Mathematics, University of Oxford, United Kingdom (OX2 6GG)

7. Center for Social Sciences, Southern University of Science and Technology, Shenzhen, China (518055)

*Corresponding author: [ruiliang.liu@arch.ox.ac.uk](mailto:ruiliang.liu@arch.ox.ac.uk); liucheng@nwu.edu.cn

1. **A general background introduction of Anyang and the key metal assemblages**

Anyang, or Yinxu (Yin ruins) is the last capital of the late Shang (ca. 1300 to 1050 BCE). Located in the suburb of modern Anyang city, Henan province, its area is approximately 24 km^2^. The Huan River divides it into two parts. Within this large area were distributed a variety of differing sites, including settlements, aggregated royal tombs, temples, palaces, family cemeteries and workshops^1,2^.

The mainstream chronological framework is to divide the entire material assemblage at Anyang into four time periods, or four phases that can be traced back to 1950-1960s^3,4^. The principal approach is integration of the stratigraphic order and the typological variation. The two major materials employed are pottery and bronze. It has become the standard framework and language to report and describe past and new excavations. Materials from previous well-known excavations are often taken as the criteria to anchor new discoveries into one of the four phases. The study of oracle bones and radiocarbon dating also contribute to this four-phase chronology. Scholars have even correlated the lineage of Anyang kings to these four phases (Table S1 and Fig. S1).

An impressive number of building foundations have been identified as palaces and temples in a village named Xiaotun, occupying up to 35,000 m^2^ ^1^. The other most common discovery is the astonishing number of cemeteries, which are widespread in the modern city of Anyang. Most of the royal cemeteries were discovered in Xibeigang, north of Anyang, except one huge tomb in Wuguan village, and those found in Xiaotun, including the tomb of Fu Hao. Although those in Xibeigang have been severely looted, probably multiple times, through comparison of their sizes relative to that of Fu Hao, the only one found intact in Anyang and buried with 1.6 ton of metal, there is no doubt that the content initially dedicated to those kings must have been enormous.

The discovery of bronze workshops sheds more light on various aspects of metal production at Anyang. So far, they have been found in many places within Anyang, including North Miaopu, Northeast Xiaotun, Xiaomintun, Dasikong and Xuejiazhuang^1,5^. The most convincing evidence for bronze workshops is the discovery of broken moulds, fragmented pieces of crucibles, burnt ground surface and various tools. The largest site is at North Miaopu, which covered an area greater than 10,000 m^2^. Some sites were excavated before the Sino-Japanese war (1937-1945) and the finds were moved to Taiwan in 1949. Those found after the war are kept in the Institute of Archaeology (Academy of Social Science, IA CASS). Li Yung-ti has thoroughly synthesized both old and new material relating to these bronze workshops^5^. He argued that while the archaeological data is rather fragmented, it is still possible to see two different forms of foundry organization, holistic and prescriptive. A holistic form of production indicates special types of objects manufactured by a small group of individuals who are capable of finishing the entire procedures. By contrast, a prescriptive one requires subtler labour division. Those who are specialized in one unit of production may not be skilled in the next, so they have to pass down the semi-finished products for further processing. He meticulously compared the archaeological remains in the bronze foundries in Anyang and argued that the foundry in Xiaotun, though is relatively smaller in size, has a variety of remains corresponding to a full range of foundry activities and object types (e.g. vessels, weapons and chariots), and appears consistent with the holistic form. In contrast, the foundry debris unearthed in Xiaomintun Southeast is ten times larger than the one in Xiaotun but overwhelmingly focused on the production of vessels. More importantly, Xiaomintun Southwest seemed only capable of casting and finishing bronzes. No clear evidence can be found in situ in favour of the existence of clay processing, mould making or other activities. Therefore, Li suggested a prescriptive form of production was undertaken at Xiaomintun Southwest.

A variety of fine materials such as jade, white pottery and ivory, and human/animal sacrifices, together with these bronze ritual vessels in tombs constitute the complete ritual paraphernalia, which reflect the socio-political rankings of their owners. Ancestor worshipping played a vital role during the late Shang period and bronze ritual vessels are central to this belief as they were employed to offer food and drink to the ancestors.

Whilst a large number of tombs have been excavated with bronzes, only a small fraction has been chemically analysed. Fortunately, these finds cover almost all major phases of Anyang (II-IV). The tomb content demonstrates the discrepancy in social status. In addition to bronze ritual vessels, these considered to be tombs of the high elites in this article for the analysis, contain a considerable number of invaluable objects of different materials (Table S2). No comparable example can be found in the Western Area, even though some of which contain several pieces of bronzes, hence they have been categorized as low elites.


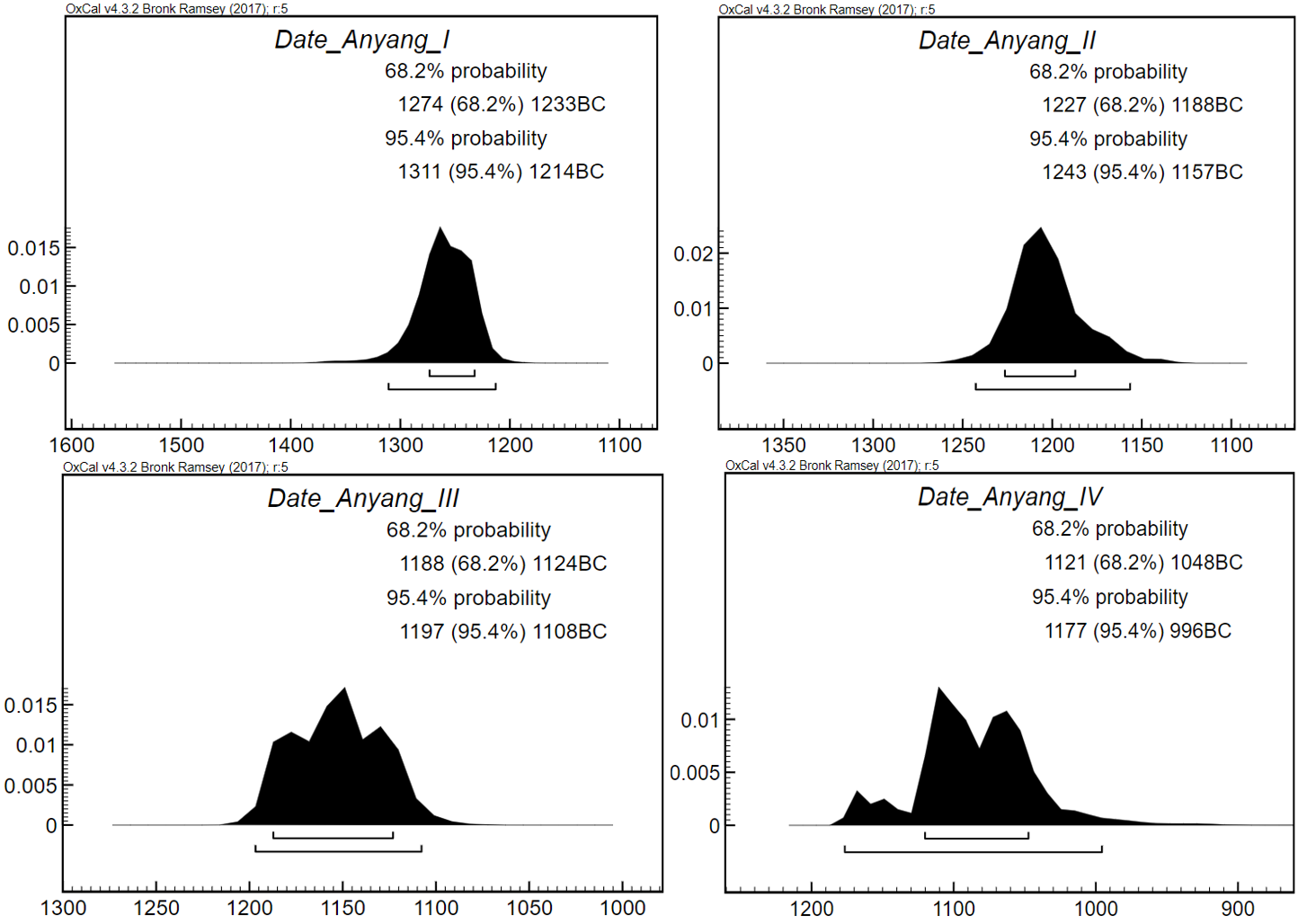


Fig. S1 Modelled radiocarbon dating results for the Anyang four phases (performed in OxCal 4.3 with IntCal13, detailed code to be found in section 4)

Table S1 Summary of various opinions on Anyang four chronological phases and its correlation with the list of kings

|  | ***Before Pangeng*** | ***Pangeng, Xiaoxin, Xiaoyi*** | | ***Wuding, Zujia, Zuyi*** | | | | ***Lingxin, Kangding, Wuyi, Wending*** | | | ***Diyi, Dixin*** |
| --- | --- | --- | --- | --- | --- | --- | --- | --- | --- | --- | --- |
| Zouheng (1964) |  |  | | **Yinxu I phase** | **Yinxu II phase** | | | **Yinxu III phase** | | | **Yinxu IV phase** |
|  |  |  | | YM232, YM333 | YM388, YM188, YM331, Wuguan 59M1, Wuguan Big Tomb, HPKM1001, Sikong M157 | | | YM238, HPKM1002, Sipanmo M8, **Fuhao M5**, Sikong M304, Gaolouzhuang M8 | | | Sipanmo M6, Sipanmo M4, Sikong M239, HPK M2020, HPKM1003, HGH10 |
| Zhang (1997) |  | YM232, YM333, YM388, YM188, YM331 | | | | | Wuguan Big Tomb, **Fuhao M5**, YM238, HPKM1002, HPKM1400 | | Sikong M51, Sikong M53, Gaolouzhuang M8, HGH10 | | |
| Zheng and Chen (1985) |  | **Yinxu I phase** | | **Yinxu II phase** | | | | **Yinxu III phase** | | | **Yinxu IV phase** |
|  |  | Early | Late |  |  |  |  |  |  |  |  |
|  |  | YM232, YM333, YM388, SJM1, M3, J1 | Wuguan 59M1, YM331 | Wuguan Big Tomb, YM238, YM188, **Fuhao M5**, Xiaotun M17, Xiaotun M18 | | | | GM2508,  PNM172 | | | GM2579, GM1713, 82Xiaotun M1, Sikong M53 |
| Yang and Yang (1985) |  | **Yinxu I phase** | **Yinxu II phase** | | | | | | | | **Yinxu III phase** |
|  |  |  | Early | Middle | | | | Late | | |  |
|  |  | YM232, YM333, YM388, YM331, SJM3 | YM188, 59Wuguan M1, Xiaotun 73H13 | **Fuhao M5**, Xiaotun M17, Xiaotun M18, GM613 | | | | PNM172, GM198, GM907, SikongM51 | | | GM269, GM284, GM793, GM1053, GM1573, Sikong M53, GM1713 |
| Zhu (2009) |  | YM232, YM333, YM388, Sanjiazhuang M3 | YM331, YM188, 59Wuguan M1 | **Fuhao M5**, Xiaotun 17, Xiaotun M18, Sikong M539, 83Sikong M663, GM613, Xuejiazhuang M3, PNM105, GM161 | | | | 57Gaolouzhuang M8, SikongM51, PNM172, Qijiazhuang, M269, **Guojiazhuang M160** | | | Sikong M53, GM269, GM2579, GM1713, 83GuoM1, Guojiazhuang North M6, 82Xiaotun M1 |
| Yue (2004) | **Yinxu I phase** | | | **Yinxu II phase** | | | | **Yinxu III phase** | | | **Yinxu IV phase** |
|  |  |  |  | Early | | Late | | Early | | Late |  |
|  | YM232, YM333, YM388, Sanjiazhuang M3, J1, 99AHDM10 | | | YM331, YM188, 59 WuM1, Xiaotun 73H13, some bronzes in Fuhao tomb | | **Fuhao M5**, Xiaotun M17, Xiaotun M18, Siokong M539, 83 Sikong M663, YM238, 95GuoM26, Huayuanzhuang M54 | | Qi M269, 63PNM172, GM2508, GM907 | | **Guojiazhuang M160**, GM198, Sikong M51, 82Miaopu M54 | **North Liujiazhuang M1046**, 82Xiaotun M1, GM1713, Guojiazhuang North M6, Liujiazhuang North M9, Sikong M53, Qijiazhuang M63, Qijiazhuang East M231 |
| Huang (2013) |  | | | Fuhao tomb,  Huayuanzhuang M54, Xiaotun M18, Xiaotun M238, Guojiazhuang M26, North Liujiazhuang M793, Wuguan W8, 80 Dasikong M539, 83 Dasikong M663, 86 Dasikong M29, South Miaopu M58, 1957 Gaokouzhuang M58, | | | | Guojiazhuang M160, Eastern Qijiazhuang M269, 1958 Dasikong M51, 1957 Xuejiazhuang M8, North Liujiazhuang M637, Dasikong M101, Tijiakou M3 | | | Dasikong M303,  North Liujiazhuang M1046, Angang Wu Shenghuoqu M6, 86 Guojiazhuang M6, 83 Liujiazhuang M9, Western Area M1713, 58 Dasikong M51, 58 Dasikong M53, Western Area M2579, 1982 Miaopu M41, Qijiazhuang M41, Qijiazhuang M63, Qijiazhuang M231, Qijiazhuang M235, Guojiazhuang M53, Eastern Dasikong M7, Western Area M07, Xiaomintun M17, South Liujiazhuang M66 |

Table S2 Summary of the size, structure and content of the high-elite tombs at Anyang


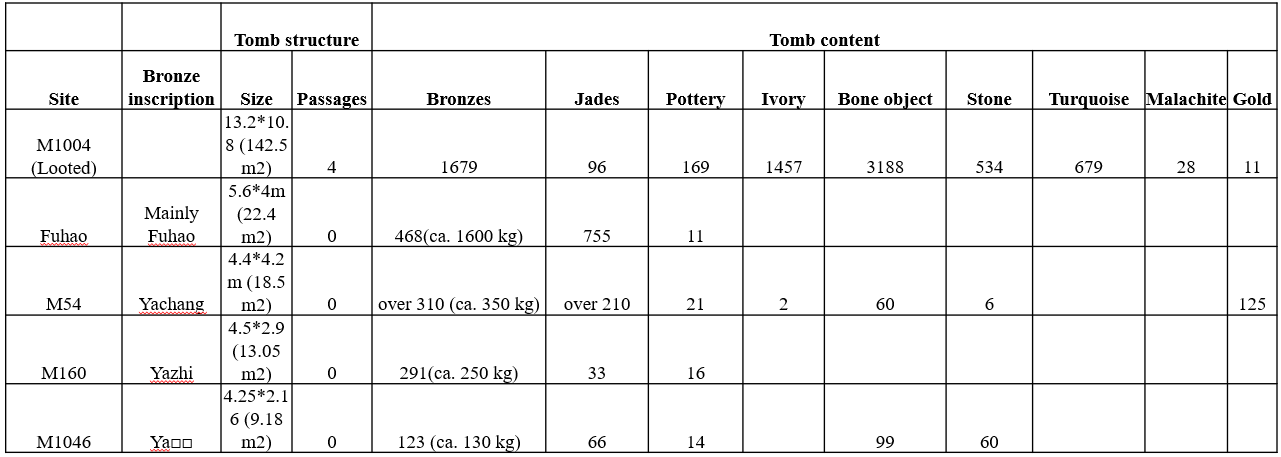


**2. Statistical analysis**

2.1 KS test

The Kolmogorov–Smirnov test (KS test) is performed in software R (version 3.6.2, package ks.test). The primary reason to choose KS test is that it is non-parametric and does not assume normal distribution in the original datasets. It also allows scholars to compare datasets in different sizes^6^. The p values have been listed in Table S3. All the p values greater than 0.05 are highlighted and they suggest that the level of tin or lead in the two assemblages of interests are statistically indistinguishable. For instance, no evidence can suggest that the distribution of tin in M1004 weapons appears as the same as that of Western Area Phase II vessels.

Table S3 P-values of KS test (p values greater 0.05)


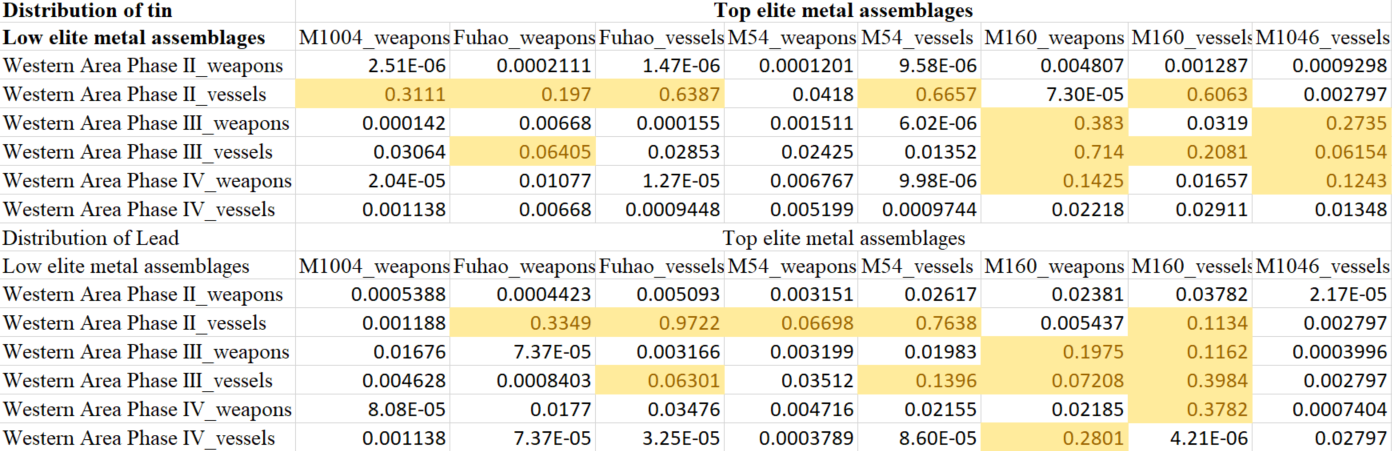


2.2 Kernel density analysis

While Fig. 2-3 in the main text present data in the form of skyline plots (based on histograms) is accessible, it is subject to a number of issues including the unit sum problem and the lack of robust statistical tests^6^. To overcome these issues, a kernel density rendition of the plot of the ratios of Sn/Cu and Pb/Cu in each object has been conducted, which compares the alloying patterns in vessels and weapons from high elite tombs (upper row) and low elite tombs (lower row) from Phases II, III and IV (columns from left to right). The KDE matrix is based on the pair-wise overlap between two distributions^7^. Compared to common methods such as box-and-whisker, it enables scholars to compare two variables together in a quantitative way. It produces a ratio which measures the likelihood of assemblage Y being a sub-set of assemblage X (which is not the same as the likelihood of X being a sub-set of Y). This value should approach 1 if the distribution of the two datasets is similar, but could exceed 1 if dataset Y is located on top of the densest area of dataset X. For instance, Fuhao’s vessels clearly overlap with the Phase II Low Elite Vessels (entry B-I in KDE matrix = 1.3837), but the reverse (I-B = 0.8333) is much less significant (Table S4). This means the variation of tin and lead in the Fuhao vessels is very likely to be a subset of what is represented by the Phase II Lower Class vessels. The reason is that the Fuhao vessels have a very tight distribution in Sn/Cu – Pb/Cu space than Phase II Low Elite Vessels, showing a clear contrast between primary practice and secondary alloying. In addition, Fig. S2 shows that the broad variations of alloying in both ritual vessels and weapons of the low-elites, particularly in the third and fourth phase, imply a relatively higher probability of mixing and recycling.
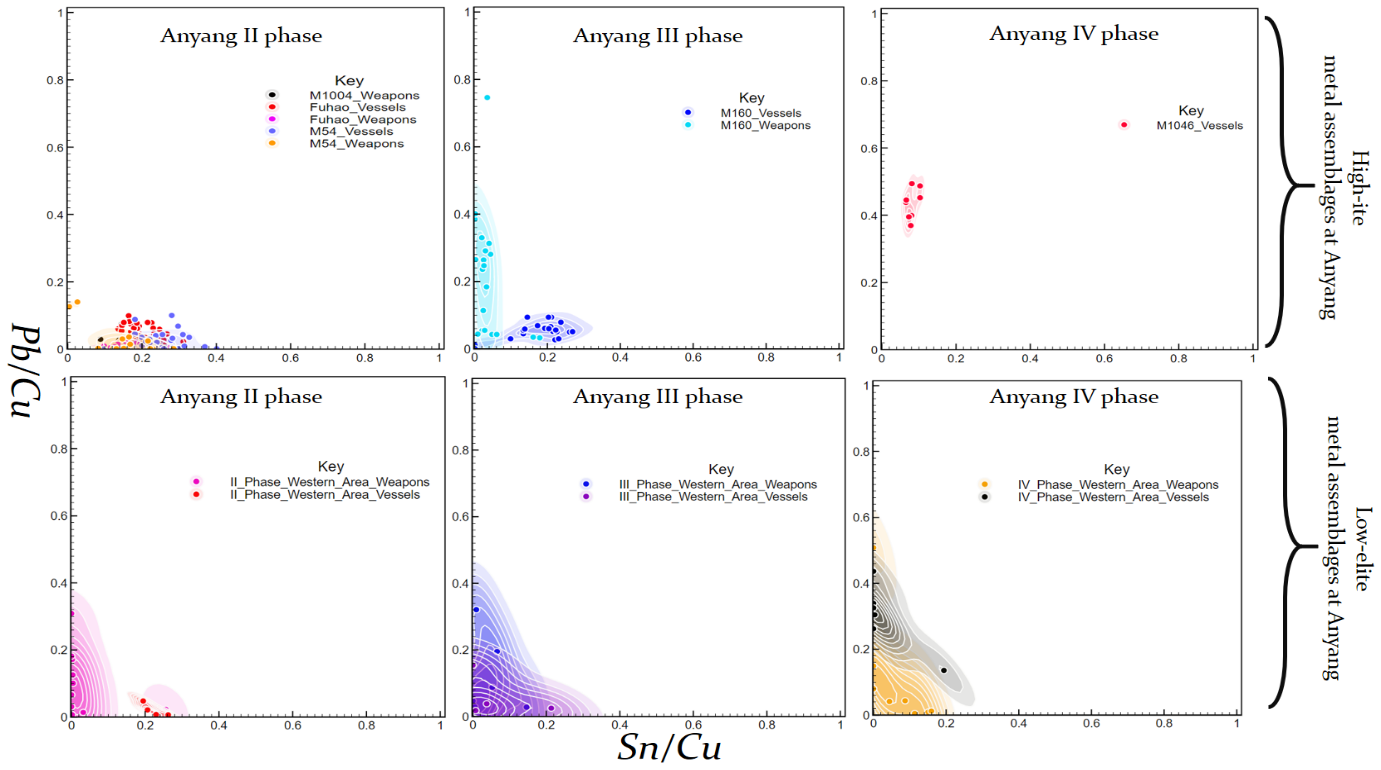


Figure S2 Kernel density estimation of tin and lead in Anyang metal objects

Table S4. Results of kernel density estimation for the comparative study between high/low elite metal assemblage

|  | A | B | C | D | E | F | G | H | I | J | K | L | M | N |
| --- | --- | --- | --- | --- | --- | --- | --- | --- | --- | --- | --- | --- | --- | --- |
| A-M1004-Weapons | 1.0000 | 1.0359 | 0.9047 | 0.7769 | 1.5973 | 0.2206 | 0.0568 | 0.0000 | 0.2318 | 0.2777 | 3.0509 | 0.6067 | 0.1102 | 0.8319 |
| B-Fuhao-Vessels | 0.3782 | 1.0000 | 0.7455 | 1.1246 | 1.1243 | 0.5425 | 0.0349 | 0.0000 | **1.3837** | 0.2267 | 2.8264 | 0.3846 | 0.2043 | 0.5521 |
| C-Fuhao-Weapons | 0.2066 | 0.7294 | 1.0000 | 0.9244 | 1.2064 | 0.2000 | 0.0742 | 0.0000 | 1.2200 | 0.3068 | 2.8070 | 0.5681 | 0.1057 | 0.7997 |
| D-M54-Vessels | 0.0847 | 0.6968 | 0.3644 | 1.0000 | 0.6080 | 0.3647 | 0.0209 | 0.0000 | 1.2523 | 0.1512 | 1.8576 | 0.1605 | 0.1592 | 0.2640 |
| E-M54-Weapons | 0.2115 | 0.5193 | 0.4024 | 0.5693 | 1.0000 | 0.2687 | 0.2200 | 0.0000 | 0.5345 | 0.5122 | 3.0068 | 0.8942 | 0.0793 | 1.0661 |
| F-M160-Vessels | 0.0030 | 0.4089 | 0.0019 | 0.5185 | 0.3543 | 1.0000 | 0.0716 | 0.0000 | 0.5379 | 0.4195 | 2.7127 | 0.6263 | 0.2469 | 0.5233 |
| G-M160-Weapons | 0.0025 | 0.0310 | 0.0000 | 0.0348 | 0.1226 | 0.1023 | 1.0000 | 0.0002 | 0.0165 | 0.8119 | 1.4508 | 1.6274 | 0.9066 | 1.2161 |
| H-M1046-Vessels | 0.0000 | 0.0000 | 0.0000 | 0.0000 | 0.0000 | 0.0000 | 0.2722 | 1.0000 | 0.0000 | 0.0163 | 0.0000 | 0.1170 | 0.1946 | 0.2062 |
| I-II Phase Western Area-Vessels | 0.0339 | **0.8333** | 0.3410 | 1.0474 | 0.7039 | 0.5343 | 0.0146 | 0.0000 | 1.0000 | 0.1320 | 2.0265 | 0.1493 | 0.1927 | 0.2521 |
| J-II Phase Western Area-Weapons | 0.0034 | 0.0851 | 0.0137 | 0.1947 | 0.1277 | 0.1573 | 0.6862 | 0.0000 | 0.1171 | 1.0000 | 2.4146 | 1.5606 | 0.2586 | 1.1421 |
| K-III Phase Western Area-Vessels | 0.0067 | 0.4725 | 0.0056 | 0.4616 | 0.3516 | 0.2974 | 0.3617 | 0.0000 | 0.8680 | 0.6524 | 1.0000 | 1.0581 | 0.0746 | 0.7666 |
| L-III Phase Western Area-Weapons | 0.0119 | 0.0855 | 0.0000 | 0.0832 | 0.2773 | 0.1840 | 0.5654 | 0.0000 | 0.0005 | 0.8854 | 2.1326 | 1.0000 | 0.5244 | 0.9886 |
| M-IV Phase Western Area-Vessels | 0.0000 | 0.0000 | 0.0000 | 0.0003 | 0.0000 | 0.0006 | 0.7704 | 0.0000 | 0.0000 | 0.1846 | 0.1194 | 0.6324 | 1.0000 | 0.5770 |
| N-IV Phase Western Area-Weapons | 0.1264 | 0.1071 | 0.2777 | 0.0968 | 0.5028 | 0.2000 | 0.4869 | 0.0000 | 0.0000 | 1.0381 | 2.8604 | 1.3740 | 0.0240 | 1.0000 |

Table S5 Summary of analytical techniques


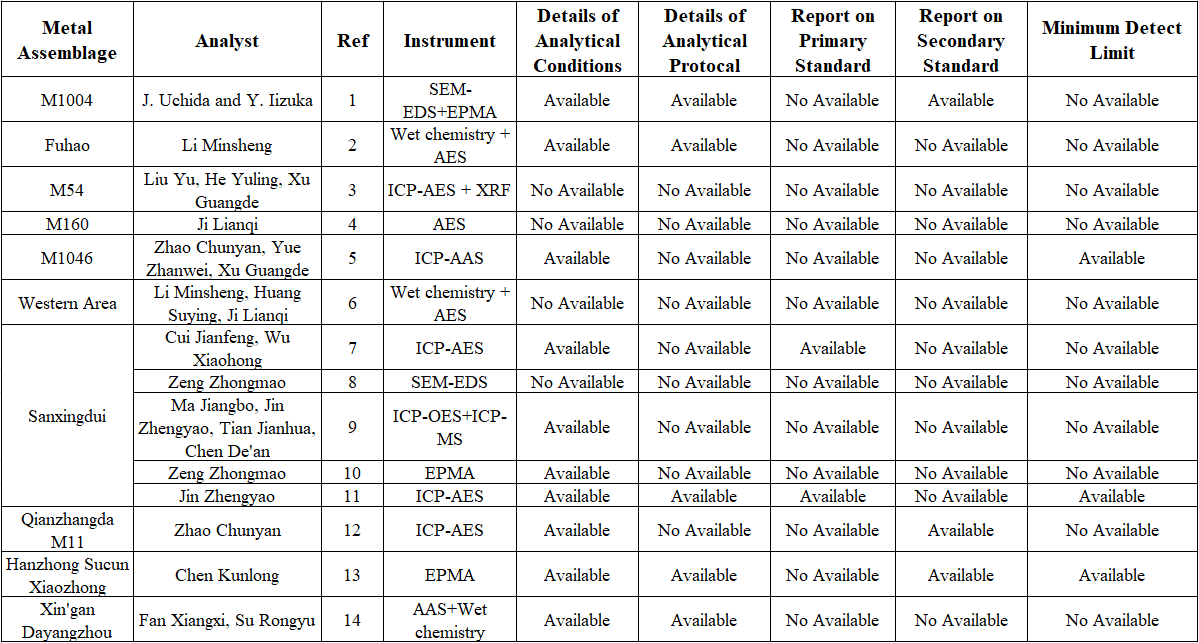


Reference for Table S5

1 Uchida, J., and Y. Iizuka, 2015, The metallurgical study of the Yinxu bronzes collected by the Academic Sinica, in Celebrating the eighty anniversary of discovering Anyang, 81-112, Y.-t. Li, ed., Institute of history and philology, Academia Sinica, Taipei.

2 Li, M., 1982, Report one on the bronzes at Yinxu—the Fu Hao tomb, Kaoguxue jikan, 2(2), 181-193 (in Chinese).

3 Liu, Y., Y. He, and G. Xu, 2007, The scientific analysis of bronzes in tomb M54 and M60, in The Shang tombs of Eastern Huayuanzhuang, Anyang, 289-296, IA CASS, ed., Science Press, Beijing (in Chinese).

4 Ji, L., 1997, The study of the chemical compositions of copper objects from M160 of Guojiazhuang, Anyang, Henan, Kaogu, (2), 80-84 (in Chinese).

5 Zhao, C., Z. Yue, and G. Xu, 2008, Chemical analysis of bronzes of M1046 at North Liujiazhuang of Anyang, Wenwu, (1), 92-94 (in Chinese).

6 Li, M., S. Huang, and L. Ji, 1984, Report two on the bronzes at Yinxu—the Fu Hao tomb, Kaoguxue jikan, (4), 328-375 (in Chinese).

7 Cui, J., and X. Wu, 2013, Metallurgical and lead isotopic analysis of some bronzes at Sanxingdui—some new understandings of Sanxingdui culture, Nanfang minzu kaogu, (9), 237-250 (in Chinese).

8 Zeng, Z., 1991, Chemical analysis of bronzes from the Pit II at Sanxingdui, Guanghan, Sichuan wenwu, (1), 72-74 (in Chinese).

9 Ma, J., Z. Jin, J. Tian, and D. Chen, 2012, The alloying composition and metallographic study of Sanxingdui bronzes, Sichuan wenwu, 2, 90-96 (in Chinese).

10 Zeng, Z., 1989, Chemical analysis of bronzes from the Pit II/II at Sanxingdui, Guanghan, Sichuan wenwu, (s1), 76-80 (in Chinese).

11 Jin, Z., 2008, Lead isotope archaeology in China, Press of University of Science and Technology, Beijing (in Chinese).

12 Zhao, C., 2005, Chemical analysis of the bronzes of Qianzhangda site, in Tengzhou Qianzhangda Cemetery, Institute of Archaeology Chinese Academy of Science, ed., Wenwu press, Bejing.

13 Chen, K., 2009, Scientific Study on the Shang Dynasty Bronzes Unearthed from Hanzhong, Shaanxi Province: Materials and Manufacturing Techniques, University of Science and Technology Beijing (in Chinese).

14 Fan, X., and R. Su, 1997, The alloying composition of bronzes in the Shang tomb of Xin’gan) in Xin’gan Dayangzhou Shang tomb, 241-244, Jiangxi Provincial Museum, Jiangxi Institute of Archaeology, and Xin'gan Museum, eds., Wenwu press, Beijing (in Chinese).

1. **OxCal code for Figure 1 in the online supplementary material (performed in OxCal 4.3 https://c14.arch.ox.ac.uk/oxcal.html)**

Options()

{

Resolution=5;

};

Plot()

{

Outlier_Model("General",T(5),U(0,4),"t");

Sequence ()

{

After("Start Anyang", calBP(3500));

Date ("Start_Anyang_I");

Date ("End_Anyang_I");

Date ("Start_Anyang_II");

Date ("End_Anyang_II");

Date ("Start_Anyang_III");

Date ("End_Anyang_III");

Date ("Start_Anyang_IV");

Date ("End_Anyang_IV");

After("End Anyang", calBP(2000));

};

Sequence ()

{

Boundary ("=Start_Anyang_I");

Phase ("Anyang_I")

{

R_Date(" ZK5586 ", 3030 , 35 )

{

Outlier("General", 0.05);

};

R_Date(" ZK5595 ", 3039 , 42 )

{

Outlier("General", 0.05);

};

R_Date(" ZK5501 ", 2920 , 35 )

{

Outlier("General", 0.05);

};

R_Date(" SA99101 ", 3105 , 34 )

{

Outlier("General", 0.05);

};

R_Date(" SA98169_2 ", 3063 , 34 )

{

Outlier("General", 0.05);

};

R_Date(" SA.98187 ", 3039 , 35 )

{

Outlier("General", 0.05);

};

R_Date(" SA98160 ", 2977 , 42 )

{

Outlier("General", 0.05);

};

R_Date(" SA98I61 ", 2994 , 41 )

{

Outlier("General", 0.05);

};

R_Date(" SA98162 ", 2983 , 55 )

{

Outlier("General", 0.05);

};

Date ("Date_Anyang_I");

};

Boundary ("=End_Anyang_I");

Boundary ("=Start_Anyang_II");

Phase ("Anyang_II")

{

R_Date(" ZK5511 ", 2964 , 33 )

{

Outlier("General", 0.05);

};

R_Date(" ZK5523 ", 2994 , 37 )

{

Outlier("General", 0.05);

};

R_Date(" ZK5521 ", 2908 , 32 )

{

Outlier("General", 0.05);

};

R_Date(" SA98181 ", 2989 , 42 )

{

Outlier("General", 0.05);

};

R_Date(" SA99040_2 ", 2945 , 48 )

{

Outlier("General", 0.05);

};

R_Date(" SA99094 ", 3023 , 32 )

{

Outlier("General", 0.05);

};

R_Date(" SA98218 ", 2985 , 32 )

{

Outlier("General", 0.05);

};

Date ("Date_Anyang_II");

};

Boundary ("=End_Anyang_II");

Boundary ("=Start_Anyang_III");

Phase ("Anyang_III")

{

R_Date(" ZK5578 ", 2937 , 33 )

{

Outlier("General", 0.05);

};

R_Date(" ZK5579 ", 2962 , 35 )

{

Outlier("General", 0.05);

};

R_Date(" ZK5581 ", 2960 , 37 )

{

Outlier("General", 0.05);

};

R_Date(" ZK5582 ", 2888 , 35 )

{

Outlier("General", 0.05);

};

R_Date(" ZK5587 ", 2856 , 35 )

{

Outlier("General", 0.05);

};

R_Date(" ZK5588 ", 2956 , 35 )

{

Outlier("General", 0.05);

};

R_Date(" ZK5590 ", 2935 , 35 )

{

Outlier("General", 0.05);

};

R_Date(" ZK5592a ", 2946 , 35 )

{

Outlier("General", 0.05);

};

R_Date(" ZK5525 ", 2882 , 37 )

{

Outlier("General", 0.05);

};

R_Date(" ZK5543 ", 2983 , 34 )

{

Outlier("General", 0.05);

};

R_Date(" ZK5538 ", 2954 , 37 )

{

Outlier("General", 0.05);

};

R_Date(" ZK5529 ", 2951 , 35 )

{

Outlier("General", 0.05);

};

R_Date(" ZK5534 ", 2870 , 35 )

{

Outlier("General", 0.05);

};

R_Date(" SA9821D ", 2996 , 44 )

{

Outlier("General", 0.05);

};

R_Date(" SA98219 ", 3005 , 32 )

{

Outlier("General", 0.05);

};

R_Date(" SA98227-2 ", 2961 , 34 )

{

Outlier("General", 0.05);

};

R_Date(" SA98166 ", 2913 , 45 )

{

Outlier("General", 0.05);

};

R_Date(" SA98251 ", 2921 , 35 )

{

Outlier("General", 0.05);

};

Date ("Date_Anyang_III");

};

Boundary ("=End_Anyang_III");

Boundary ("=Start_Anyang_IV");

Phase ("Anyang_IV")

{

R_Date(" ZK5572 ", 2942 , 35 )

{

Outlier("General", 0.05);

};

R_Date(" ZK5551 ", 2912 , 31 )

{

Outlier("General", 0.05);

};

R_Date(" ZK5559 ", 2900 , 35 )

{

Outlier("General", 0.05);

};

R_Date(" ZK5558 ", 2892 , 35 )

{

Outlier("General", 0.05);

};

R_Date(" ZK358 ", 2932 , 34 )

{

Outlier("General", 0.05);

};

};

Date ("Date_Anyang_IV");

Boundary ("=End_Anyang_IV");

};

};

**Reference**

1 IA CASS. *Discovery and study of Yinxu*. (Science Press, 1994).

2 Li, J. *Anyang*. (Dawson, 1977).

3 Zou, H. Preliminary discussion on the chronoligcal phases of Yinxu. *Beijing daxue xuebao*, 37-63 (1964, in Chinese).

4 Zou, H. Preliminary discussion on the chronoligcal phases of Yinxu (Part II). *Beijing daxue xuebao*, 63-90 (1964, in Chinese).

5 Li, Y.-t. *The Anyang bronze foundries: archaeological remains, casting technology and production organization*, Harvard University, (2003).

6 Pollard, A. M., Liu, R., Rawson, J. & Tang, X. From alloying composition to alloying practice: Chinese bronzes. *Archaeometry* **61**, 70-82 (2019).

7 Bronk Ramsey, C., Housley, R. A., Lane, C. S., Smith, V. & Pollard, A. M. The RESET tephra database and associated analytical tools. *Quaternary Science Reviews* **118**, 33-47 (2015).
